# The effect of dye-sensitized solar cell based on the composite layer by anodic TiO_2_ nanotubes

**DOI:** 10.1186/1556-276X-9-671

**Published:** 2014-12-12

**Authors:** Jun Hyuk Yang, Kyung Hwan Kim, Chung Wung Bark, Hyung Wook Choi

**Affiliations:** 1 Department of Electrical Engineering, Gachon University, 1342 Seongnamdaero, Sujeong-gu, Seongnam, Gyeonggi-do, Korea

**Keywords:** DSSCs, Anodic oxidation, Photoelectrode, TiO_2_ nanotube array, Composite layer

## Abstract

TiO_2_ nanotube arrays are very attractive for dye-sensitized solar cells (DSSCs) owing to their superior charge percolation and slower charge recombination. Highly ordered, vertically aligned TiO_2_ nanotube arrays have been fabricated by a three-step anodization process. Although the use of a one-dimensional structure provides an enhanced photoelectrical performance, the smaller surface area reduces the adsorption of dye on the TiO_2_ surface. To overcome this problem, we investigated the effect of DSSCs constructed with a multilayer photoelectrode made of TiO_2_ nanoparticles and TiO_2_ nanotube arrays. We fabricated the novel multilayer photoelectrode via a layer-by-layer assembly process and thoroughly investigated the effect of various structures on the sample efficiency. The DSSC with a four-layer photoelectrode exhibited a maximum conversion efficiency of 7.22% because of effective electron transport and enhanced adsorption of dye on the TiO_2_ surface.

## Background

Dye-sensitized solar cells (DSSCs) have attracted great interest in scientific and industrial fields during the past two decades because of their low cost, impressive power conversion efficiency, and easy fabrication compared to conventional p-n junction solar cells. Despite these advantages, the low efficiency of DSSCs compared to that of silicon-based cells has limited their commercial implementation [1-4]. Consequently, there is a critical need to improve the efficiency of state-of-the-art DSSCs in order to realize next-generation solar cells. In principle, DSSCs have four components: (1) a TiO_2_ electrode film layer covered by a monolayer of dye molecules that absorbs solar energy, (2) a transparent conductive oxide layer that facilitates charge transfer from the electrode layer, (3) a counter electrode layer made of Pt or C, and (4) a redox electrolyte layer that reduces the amount of energy transferred from dye molecules [5,6]. Thus, research efforts to increase the efficiency of DSSCs have been primarily focused on improvements in the aforementioned DSSC components [7]. One of the important features of DSSCs is the mesoporous film of interconnected TiO_2_ nanoparticles (TNPs), which can supply a large surface area for the adsorption of dye molecules. However, the performance of DSSCs is limited by electron transport in the nanocrystal boundaries and recombination of electrons with the electrolyte during migration. Many researchers have reported that one-dimensional nanostructures can be used in DSSCs in place of nanoparticles to facilitate the electron transfer [8-14]. In addition to their unique electron properties, one-dimensional TiO_2_ nanostructures also function as light-scattering materials with minimal sacrifice of the surface area. On the other hand, the small specific surface area of one-dimensional nanostructures is a serious flaw as it causes insufficient dye adsorption. Achieving a balance between the two conflicting desirable features of DSSCs, a large specific surface area and an efficient electron transfer, remains a challenge.

**Figure 1:**
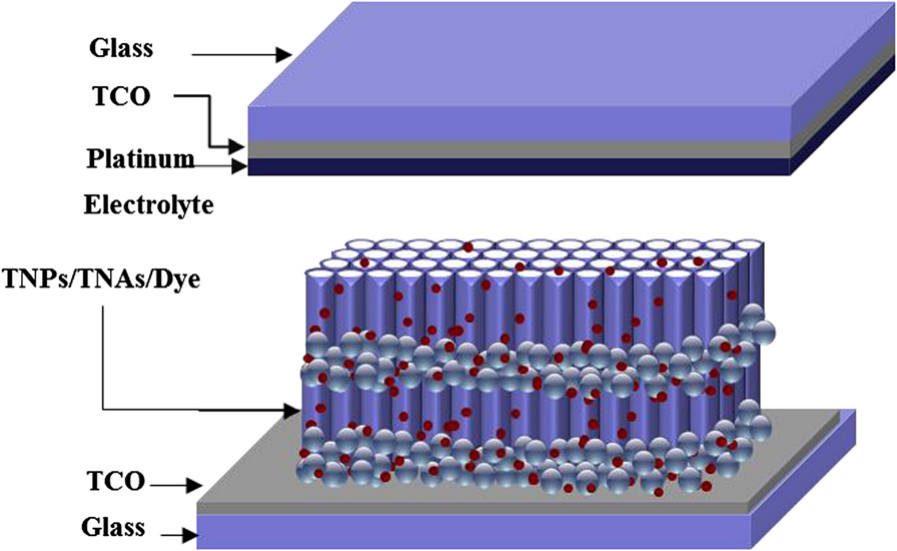
Structure of multilayer DSSCs.

In this work, we considered the aforementioned strategies in order to improve the efficiency of DSSCs. One of these approaches, involving the use of oxide semiconductors in the form of TiO_2_ nanotubes arrays (TNAs), was attempted as a novel means of improving the electron transport through the film. We fabricated a novel TNP/TNA multilayer photoelectrode via a layer-by-layer assembly process (Figure 1) and thoroughly investigated the effect of various structures on the cell efficiency.

## Methods

### Preparation of TiO_2_ nanotube array layers

**Figure 2:**
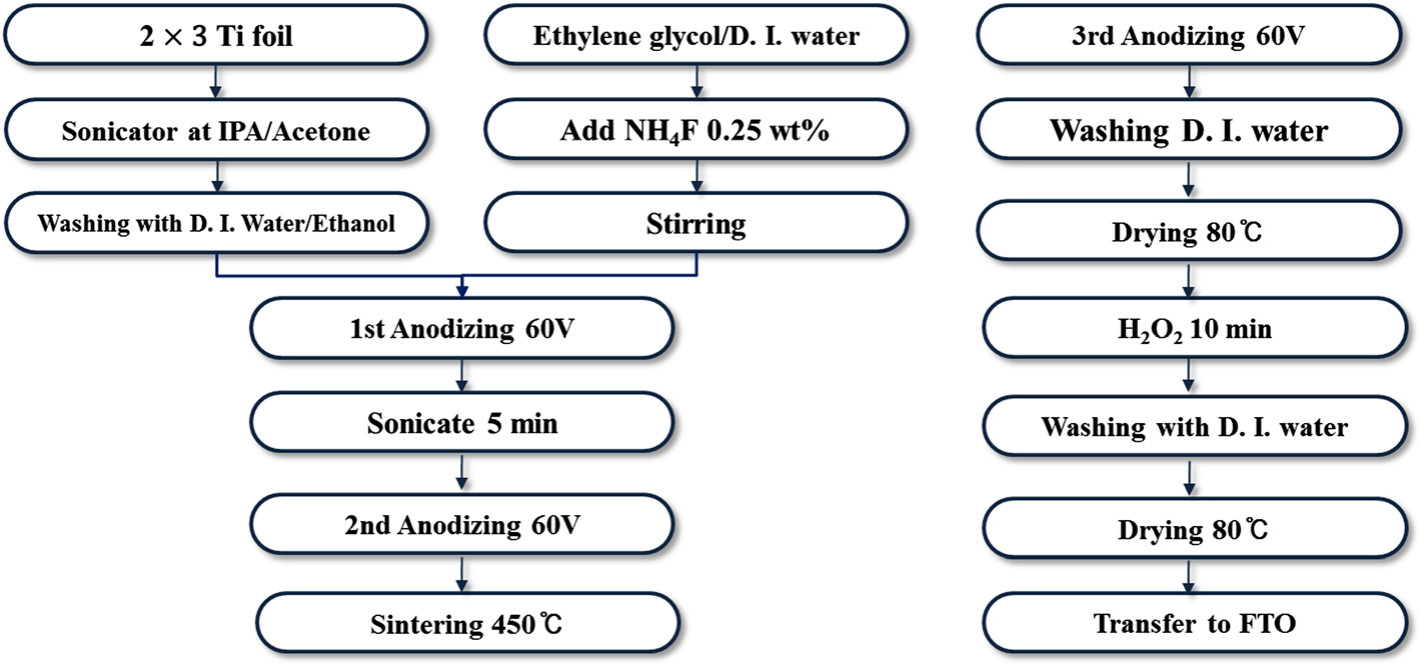
Flowchart for the manufacture of TNAs.

TNAs were prepared by an optimized three-step anodization process. A Ti foil (0.25 mm thick, 99.7% purity, Sigma-Aldrich, St. Louis, MO) with an area of 2 × 3 cm was degreased by ultrasonic agitation for 30 min each in acetone, isopropanol, and deionized water and then dried with N_2_ gas. The ethylene glycol electrolyte contained 0.25 wt.% NH_4_F (98% purity, Sigma-Aldrich, St. Louis, MO) and 2 vol.% deionized water. Anodization was performed in a two-electrode system in which the Ti foil served as the working electrode and a Pt plate (2 × 3 cm) served as the counter electrode. Anodization was conducted at room temperature at a constant voltage of 60 V for 20 min (Figure 2). Afterward, the as-prepared TNAs were removed by sonication for 5 min in methanol. The second-step anodization was done for 50 min under the same conditions. The as-prepared amorphous TNAs were crystallized into an anatase phase at 450°C for 2 h in air at a heating rate of 1°C/min. After another round of anodization for 10 min under the same conditions, followed by immersion in 30% H_2_O_2_ solution for 10 min, the anatase TNAs were detached from the Ti substrate. After rinsing and drying, the self-standing TNAs were cut into 5 × 5 mm squares for transfer onto the photoelectrode.

### Preparation of TiO_2_ layer

TiO_2_ paste was prepared from TiO_2_ powder (anatase, 99.9% purity, Sigma-Aldrich, St. Louis, MO) and used as the reference [15,16]. TiO_2_ photoelectrodes of various structures were coated on fluoride-doped tin oxide (FTO) glass using a doctor blade method (single-layer). TNAs were then transferred onto the coated photoelectrode (two-layer). The three-layer (TNP/TNA/TNP), four-layer (TNP/TNA/TNP/TNA), and five-layer (TNP/TNA/TNP/TNA/TNP) photoelectrodes were prepared by the same process. The prepared TNP/TNA photoelectrode was sintered at 450°C for 1 h in air. The Pt catalyst electrode was prepared by mixing 5 mM of H_2_PtCl_6_ in isopropyl alcohol, followed by an ultrasonic treatment. A counter electrode, which facilitates the redox reaction of the electrolyte, was fabricated by spin coating the prepared H_2_PtCl_6_ solution at 1,000 rpm for 30 s, followed by heat treatment at 450°C for 30 min.

### Assembly of dye-sensitized solar cell

The dye solution to be adsorbed on the TNP/TNA photoelectrode film was prepared by mixing 0.5 mM of Ru dye (N-719, Solaronix) with ethanol. Adsorption of the dye molecules was accomplished by placing the photoelectrode film in the dye solution and allowing it to stand in dark for 24 h. Finally, the DSSCs were fabricated by fusing the TNP/TNA photoelectrode film and the counter electrode together at 120°C for 10 min using a hot-melt sealant (60°C). The electrolyte (I^-^/I_3_^-^) was injected between the two electrodes through the inlet and then sealed with a cover glass.

### Characterization

The phases of the TNAs prepared by anodization, as well as that of TNPs, were examined by X-ray diffraction (XRD) using a Rigaku D/MAX-2200 diffractometer (Rigaku, Shibuya-Ku, Tokyo) with a Cu Kα radiation source. The morphology of the prepared TNP/TNA photoelectrode film was investigated by field-emission scanning electron microscopy (FE-SEM, S-4700, Hitachi, Chiyoda, Tokyo). The absorbance of the TNP/TNA photoelectrode film was measured using a UV-Vis spectrometer (Lambda 750, Perkin Elmer, Waltham, MA). The conversion efficiency and electrochemical impedance spectroscopy (EIS) of the fabricated DSSCs were measured using an I-V solar simulator (K3400, K3000, McScience, Youngtong, Suwon). The active area of the cell exposed to light was approximately 0.25 cm^2^ (0.5 × 0.5 cm).

## Results and discussion

**Figure 3:**
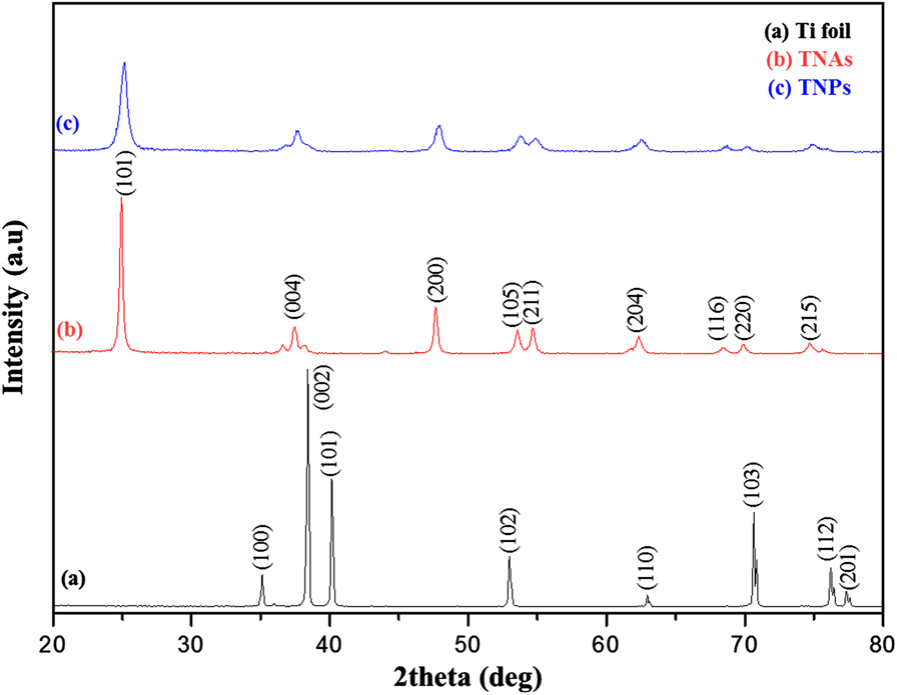
XRD patterns of (a) Ti foil, (b) TNAs, and (c) TNPs.

**Figure 4:**
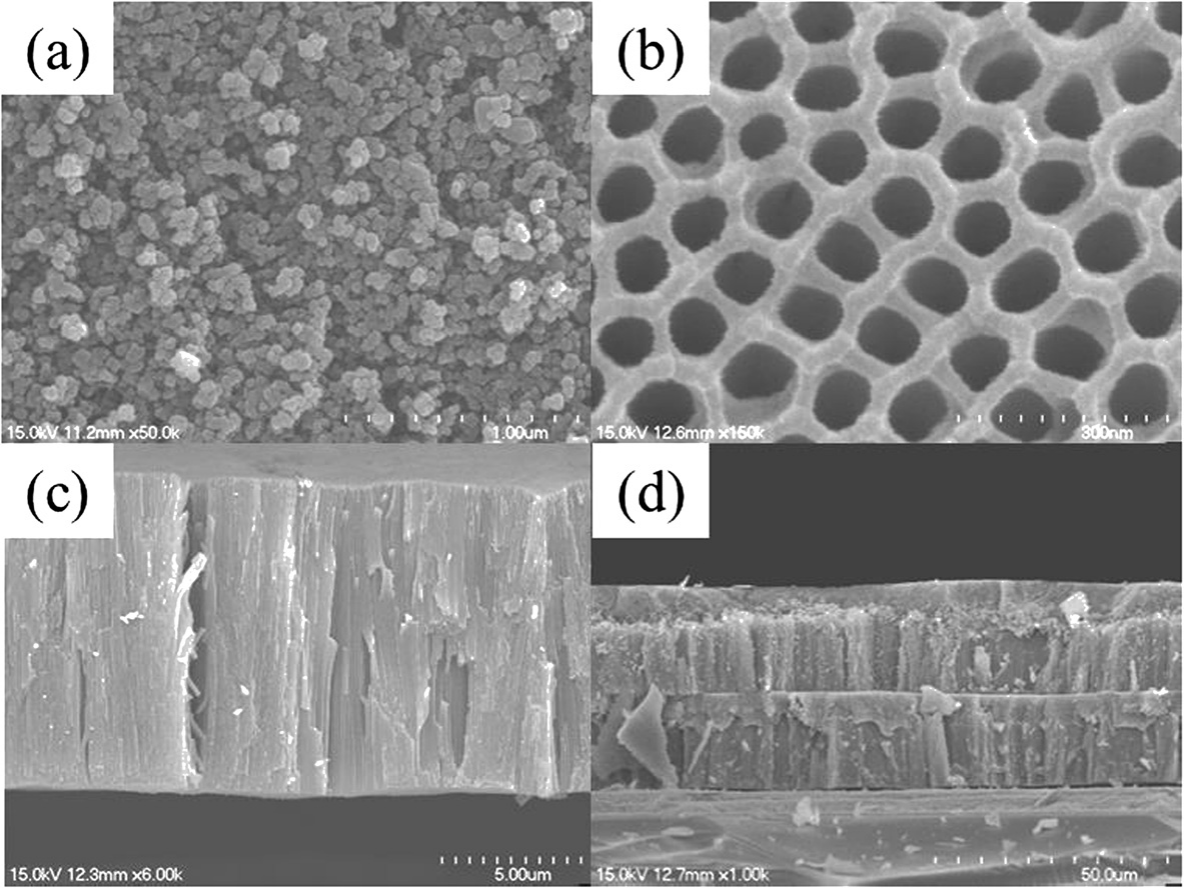
FE-SEM images of TNP and TNP surface and cross sections.

Figure 3a shows the XRD pattern of the Ti foil (JCPDS No. 44-1294). After the final anodization, the TNAs stripped from the Ti substrate were analyzed by XRD. The XRD pattern of the TNAs fabricated by calcination at 450°C (Figure 3b) shows prominent (101), (004), (200), (105), (211), (204), (116), (220), and (215) anatase peaks. Figure 3c shows the XRD pattern of the TNPs. The diffraction peaks are in good agreement with the standard JCPDS cards of anatase TiO_2_ (No. 21-1272). It is therefore preferable to produce TiO_2_ nanoparticles containing pure anatase in order to improve the DSSC efficiency [17,18].Figure 4a shows the SEM image of TNPs, wherein the particle size is approximately 20 to 30 nm. The SEM image of TNAs after the second anodization (Figure 4b) shows a uniform surface. The tube diameter is approximately 100 nm while the length can reach 10 to 13 μm with anodic oxidation under the present experimental conditions (Figure 4c). The SEM image of a cross section of the TNP/TNA multilayer photoelectrode (Figure 4d) shows relatively few cracks, a uniform tubular structure, and a vertical orientation with respect to the film surface. The TNPs were approximately 4- to 5-μm thick whereas the TNAs were approximately 10- to 13-μm thick. It is evident from Figure 4d that the TNPs, TNAs, and the substrate are well linked, which will facilitate rapid electron transport in the film.

**Figure 5:**
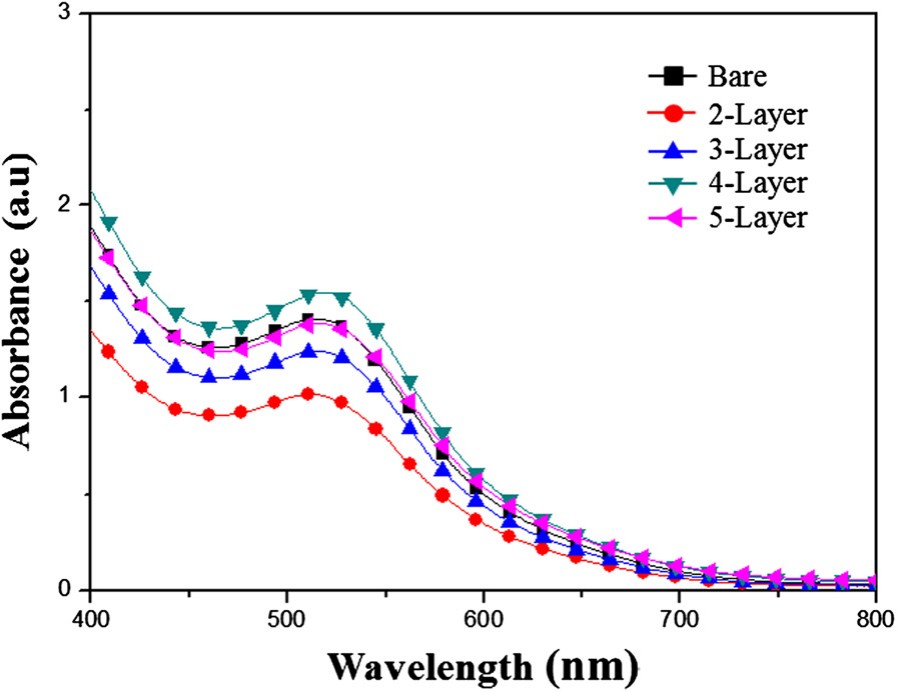
UV-Vis absorbance of single- to five-layer photoelectrodes.

Figure 5 shows the absorption spectrum of the N-719 dye in the 400- to 800-nm wavelength range in the single- to five-layer photoelectrodes. The four-layer photoelectrode shows the highest absorbance at 400 to 500 nm. According to Lambert-Beer’s law, a higher absorbance indicates a higher dye concentration. It is thus reasonable to expect a higher absorbance for the multilayer photoelectrode as it provides a larger surface area for dye adsorption than the single-layer or bare photoelectrode. Furthermore, it is known that the short-circuit photocurrent density, *J*_SC_, of DSSCs is directly correlated to the number of dye molecules. Therefore, a high number of adsorbed dye molecules results in better harvesting of incident light, and consequently, a higher *J*_SC_. On the other hand, the absorbance was observed to decrease beyond the four layers of the photoelectrode. This can be attributed to the longer distance for electron transport in thicker electrodes. The inefficient charge-transfer path increased the recombination rate of electrons, resulting in decreased photocurrent density and conversion efficiency.

**Figure 6:**
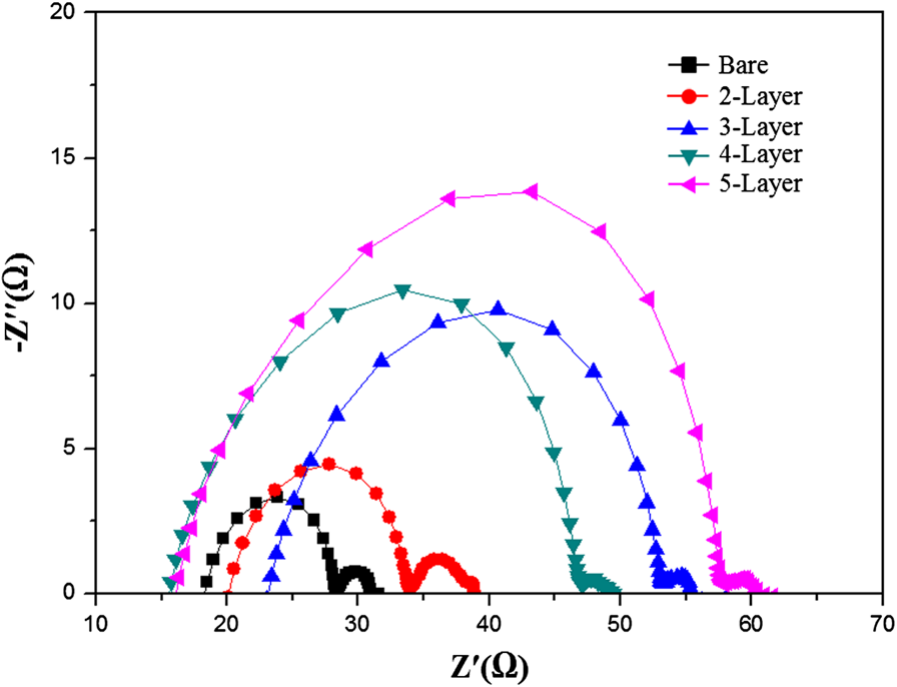
EIS Nyquist plots of DSSCs with single- to five-layer photoelectrodes.

Figure 6 shows the Nyquist plots for the single- to five-layer photoelectrodes obtained using electrochemical impedance spectroscopy (EIS). EIS measures the internal resistance and is a useful method for analyzing charge-transport processes [19]. The charge-transfer resistance increased with increase in the number of layers, as electrons will have to travel greater distances in thicker electrodes (Figure 6). Moreover, a sharp increase in the resistance was observed for the five-layer photoelectrode, which is due to the increased rate of recombination between electrons and I_3_^-^ or the oxidizing dye [20]. This is also consistent with the lower *J*_SC_ noted in the preceding discussion.

**Figure 7:**
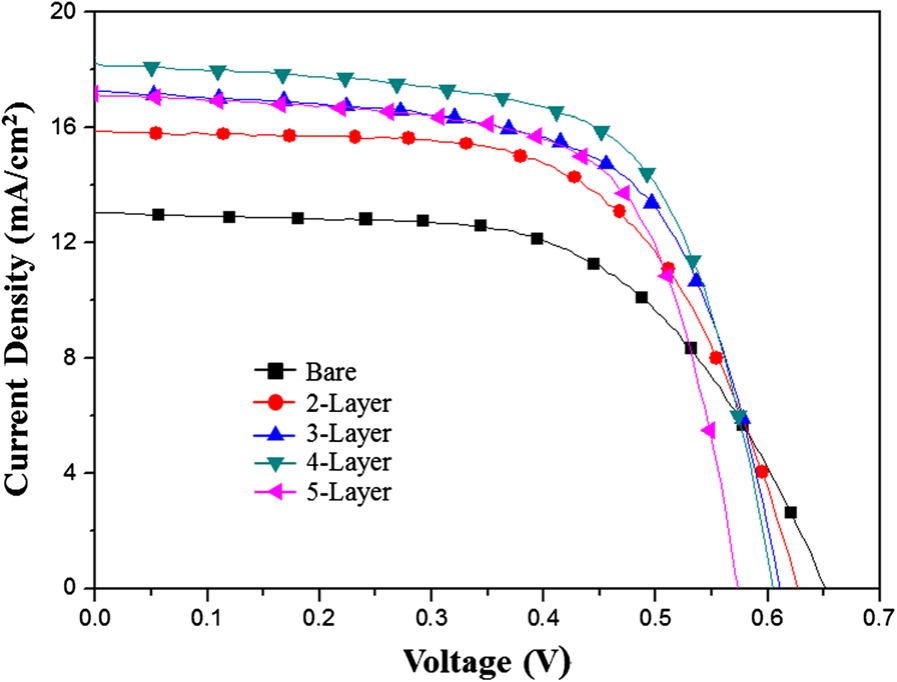
I-V curves of DSSCs with single- to five-layer photoelectrodes.

Figure 7 shows the I-V curve of the TiO_2_ film in the single- to five-layer photoelectrodes. The most important performance indicators for a solar cell are the photoelectric conversion efficiency *V*_OC_ and the fill factor FF. If the I-V curve approaches a square shape, the FF tends to become higher. A comparison of two solar cells with the same *V*_OC_ and *J*_SC_ shows that the one with a higher FF will have a stable output voltage and current and will produce more power. The photovoltaic properties of the photoelectrode films with different numbers of TiO_2_ layers are summarized in Table 1. *J*_SC_ increased with the number of TiO_2_ layers; however, beyond the fourth layer, *J*_SC_ decreased. The initial increase *J*_SC_ is due to the enhanced loading of dye molecules on the TiO_2_ film and the increase in the electron transfer rate at the TNA layer. In the case of the five-layer photoelectrode, the decrease in *J*_SC_ resulted from the increase in the charge-transfer resistance at the TiO_2_ film. FF increased from 59% in the bare photoelectrode to 65% in the four-layer photoelectrode. The DSSC fabricated using a photoelectrode with the optimum multilayer structure (i.e., four-layer photoelectrode) exhibited an efficiency of 7.22% because of the high *J*_SC_ and FF.

**Table 1:** **Integral photocurrent density (*J***_**SC**_**), open-circuit voltage (*V***_**OC**_**), fill factor (FF), and efficiency (η) of DSSCs fabricated using multilayer photoelectrodes**

**Sample**	**V**_**OC **_**(V)**	**J**_**SC **_**(A/cm**^**2**^**)**	**FF (%)**	***η *** **(%)**
Bare	0.65	13.07	59.20	5.04
Two-layer	0.62	15.88	61.76	6.14
Three-layer	0.61	17.29	63.92	6.75
Four-layer	0.60	18.22	65.51	7.22
Five-layer	0.57	17.15	66.92	6.58

## Conclusions

In this work, improvement in the performance of DSSCs by using a TNP/TNA multilayer photoelectrode was proposed. The DSSCs were constructed with TiO_2_ films made of TNAs fabricated from an anodization process and TNPs. The multilayer photoelectrode DSSCs have higher efficiencies than the single-layer or bare DSSCs. A single-layer photoelectrode DSSC with a light-to-electric energy conversion efficiency of 5.04% was achieved under a simulated solar light irradiation of 100 mW · cm^2^ (AM 1.5). The DSSCs based on a TNP/TNA multilayer photoelectrode showed a better photovoltaic performance (i.e., higher *J*_SC_ and FF) than the cell made purely of TiO_2_ nanoparticles. The conversion efficiency of DSSCs was significantly affected by the properties of TNAs. The TNP/TNA four-layer photoelectrode provided a large surface area for dye adsorption. The DSSC based on this photoelectrode was measured to have a maximum conversion efficiency of 7.22% because of effective electron transport. Thus, the use of TNAs and TNP/TNA multilayer photoelectrodes was found to be an effective method to improve the efficiency of TiO_2_ film-based DSSCs.

## Competing interests

The authors declare that they have no competing interests.

## Authors’ contributions

The first author (JHY) carried out the laboratory test, the co-authors (KHK, CWB) took charge of the small-scale laboratory measurement analysis, and the corresponding author (HWC) has controlled the whole project. All authors read and approved the final manuscript.
